# ﻿Two new species of Encyrtidae (Hymenoptera, Chalcidoidea) from the eastern Pamir Plateau, with four new records from China

**DOI:** 10.3897/zookeys.1229.138920

**Published:** 2025-02-24

**Authors:** Ouyan Xi, Shuli Zhang, Hongying Hu

**Affiliations:** 1 Xinjiang Key Laboratory of Biological Resources and Genetic Engineering, College of Life Science and Technology, Xinjiang University, Urumqi, 830046, Xinjiang, China Xinjiang University Urumqi China; 2 Key Laboratory of Biological Resources and Ecology of Pamirs Plateau in Xinjiang Uyghur Autonomous Region, College of Life and Geographic Sciences, Kashi University, Kashi, 844000, Xinjiang, China Kashi University Kashi China

**Keywords:** *Acerophagus*, *Pseudectroma*, taxonomy, alpine habitat, Xinjiang

## Abstract

Based on surveys of the superfamily Chalcidoidea (Hymenoptera) in the eastern part of Pamir Plateau from 2021 to 2023, 1770 specimens of Encyrtidae belonging to 21 species in 15 genera are identified and catalogued. Two new species, *Acerophagusalbuscorpus* Xi & Hu, **sp. nov.** and *Pseudectromastemmatosteres* Zhang & Hu, **sp. nov.**, are described and illustrated. Four species, *Ericydnusnovosibiricus* Sugonjaev & Gavrilyuk, 2012, *Copidosomacharon* Guerrieri & Noyes, 2005, *Aphycusslavai* Myartseva, 1981, and *Trichomasthusmarsus* (Walker, 1837), are newly recorded from China, and their diagnoses and illustrations are provided.

## ﻿Introduction

At present, two subfamilies, 518 known genera, and more than 4830 recognized species of Encyrtidae (Hymenoptera, Chalcidoidea) (Zhang et al. 2024) are known worldwide (UCD Community 2023; Universal Chalcidoidea Database Website. https://ucd.chalcid.org). In China, 483 species in 128 genera have been reported including 67 species in 34 genera from Xinjiang Uyhgur Autonomous Region (Zu et al. 2018; Kang et al. 2024). Most of encyrtids are primary endoparasitoids of various insects and other arthropods, but a few are hyperparasitoids (Trjapitzin 1989; Xu 1999; Zu et al. 2020). More than 400 species of Encyrtidae have been used worldwide as biological control agents against insect pests, some successfully (Hansen et al. 2012; Kapranas and Tena 2015). Species such as *Psyllaephaguspilosus* and *P.bliteus* have been successfully used for biological control of psylloids (Zou et al. 2023). *Metaphycus* Mercet are mainly primary endoparasitoids of Coccidae and Diaspididae (Hemiptera) (Tavares et al. 2019).

The Pamir Plateau, a mountain knot of the five major mountain systems in central Asia, forms the border between China and Tajikistan, Afghanistan, Pakistan, and other countries. The part on the southwestern edge of Xinjiang (36.7–39.8°N, 73.5–76.5°E), China, is commonly called the eastern (or East) Pamir Plateau. Most of the region lies at high altitude between 3300 and 6000 m above sea level (Wang et al. 2011). Its climate is characterized by a subcontinental to arid continental climate (Komatsu 2016), with average annual temperature about 3 °C with strong ultraviolet radiation. A variety of plants and insects from the Tibetan Plateau, Kashmir, Tian Shan, and Hindukush Mountain ranges converge here, and, thus, their types are complex (Nowak et al. 2022; Mętrak et al. 2023). Insects that can live in such an extreme environment need to be well adapted to it, and such environments are often represented by unique plateau species. However, records of species of Encyrtidae from such high-alpine environments are scarce worldwide and in China.

We collected specimens of Chalcidoidea in various habitats across the eastern Pamir’s main area between 2021 and 2023 using the line-transect method. After that, we carried out a more thorough taxonomic analysis of the Encyrtidae in the area using the morphometric categorization approach. We found two new species, *Acerophagusalbuscorpus* Xi & Hu, sp. nov. and *Pseudectromastemmatosteres* Zhang & Hu, sp. nov., which are desbribed here. We also found four newly recorded species in China: *Ericydnusnovosibiricus* Sugonjaev & Gavrilyuk, 2012, *Copidosomacharon* Guerrieri & Noyes, 2005, *Aphycusslavai* Myartseva, 1981, and *Trichomasthusmarsus* (Walker, 1837). Therefore, this study enriches the biodiverisity of the family Encyrtidae in the eastern Pamir and provides a reference for the application of native parasitic wasps in the Pamir Plateau.

## ﻿Materials and methods

Specimens were collected in yellow pan traps or by sweeping with a net in Atushi City, Aketao County, Wuqia County, and Tashkurgan Tajik Autonomous County, Xinjiang, China, from 2021 to 2023. Collected specimens were preserved in 99% ethanol at −20 °C (Noyes 1982). Alcohol-soaked specimens were measured under a Nikon SMZ-745T stereomicroscope and photographed with a Nikon D7000 digital camera connected to the Nikon SMZ-25 stereomicroscope. Slide-mounted specimens were examined and photographed using a Nikon Ci3 microscope. All the studied specimens were preserved in the Insect Collection of the College of Life Science and Technology, Xinjiang University, Urumqi, Xinjiang, China (**ICXU**).

Morphological terminology follows that of Noyes and Hayat (1984), Gibson et al. (1997), Trjapitzin (1989), Zhang and Huang (2004), Noyes (2010). Absolute measurements were used for body length (not including the exserted part of the ovipositor), while relative measurements were used for other dimensions. The following abbreviations are used: **POL** = minimum distance between the posterior ocelli; **OOL** = minimum distance between posterior ocellus and eye margin; **AOL** = minimum distance between posterior ocellus and anterior ocellus; **OCL** = minimum distance between posterior ocellus and occipital margin; F1–F6 = funicle segments 1–6; Gt1–7 = gastral terga 1–7.

## ﻿Results

### ﻿Species of Encyrtidae in the eastern Pamir Plateau

We obtained 1770 specimens of Encyrtidae and identified them using the keys of Trjapitzin (1989) and Noyes (2010). We recognized 15 genera and 21 species, including two new species to science, and four newly recorded species from China (Table 1). The results show that *Copidosoma* Ratzeburg, 1844 had the most specimens collected; *Copidosomatruncatellum* accounted for 79% of all specimens.

**Table 1.** Catalogue of Encyrtidae in the eastern Pamir Plateau, Xinjiang, China.

Subfamily	Genus and species	Distribution	Number
Encyrtinae	*Acerophagusalbuscorpus* Xi & Hu, sp. nov.	China (Xinjiang)	4
	*Aphycusslavai* Myartseva, 1981 *	Turkmenistan, China (Xinjiang)	11
	*Blastothrixbritannica* Girault, 1917	Palearctic, Nearctic regions	19
	*Cerchysiussubplanus* Dalman,1820	Palearctic, Oriental regions	4
	*Copidosomaagrotis* (Fonscolombe, 1832)	Palearctic region	156
	*Copidosomaaretas* (Walker, 1838)	Palearctic region	13
	*Copidosomacharon* Guerrieri & Noyes, 2005 *	Austria, Bosnia, Herzegovina, Czech Republic, UK (England), Finland, France, Norway, Spain, Sweden, China (Xinjiang)	6
	*Copidosomatruncatellum* (Dalman,1820)	Worldwide	1403
	*Copidosoma* sp.	—	56
	*Discodeskryzhanovskii* Myartseva, 1981	Turkmenistan, Uzbekistan, China	10
	*Pseudectromastemmatosteres* Zhang & Hu, sp. nov.	China (Xinjiang)	10
	*Stemmatosteresmuztagataensis* Zhang & Hu, 2023	China (Xinjiang)	4
	*Syrphophagusarundinicola* Hoffer, 1965	Armenia, Bulgaria, former Czechoslovakia, Italy, Moldova, Netherlands, Russia, China	9
	*Trichomasthusmarsus* (Walker, 1837) *	UK (England), Germany, Norway, China (Xinjiang)	8
Tetracneminae	*Anagyrusmatritensis* Mercet, 1921	Croatia, former Czechoslovakia, Iran, Azerbaijan (Nakhichevan), Spain, Tunisia, China	19
	*Charitopusfulviventris* Förster, 1860	Palearctic, Oriental, Neotropical regions	5
	*Ericydnusliaoi* Liu, Wang & Li, 2013	China (Inner Mongolia, Xinjiang)	9
	*Ericydnusnovosibiricus* Sugonjaev & Gavrilyuk, 2012*	Russia (Novosibirsk), China (Xinjiang)	8
	*Ericydnusventralis* (Dalman, 1820)	Palearctic region	9
	*Leptomastixhistrio* (Förster, 1856)	Germany, China	3
	*Rhopushanni* Zu & Li, 2020	China (Xinjiang, Tibet)	4

*Denotes a new record of the species from China.

### ﻿Descriptions of the two new species

#### ﻿ Acerophagus albuscorpus

Taxon classificationAnimaliaHymenopteraEncyrtidae

Xi & Hu sp. nov.

BD959E75-B4D7-5B80-9FE1-55E8236933C8

https://zoobank.org/72A7F982-8F98-4013-86C7-1430704BCCD9

Fig. 1

##### Type material.

***Holotype*** • ♀, in alcohol; China, Xinjiang, Tashkurgan Tajik Autonomous County, Kokyar Kirghiz Township, 38°7'50.7864"N, 74°58'39.018"E, altitude: 3565 m, 24. VII. 2021. Coll. Hu Hongying’ s insect research team by sweeping. ***Paratypes*** • 1 ♀ on slides, 2 ♀♀ in alcohol, same data as the holotype (all deposited in ICXU).

**Figure 1.**
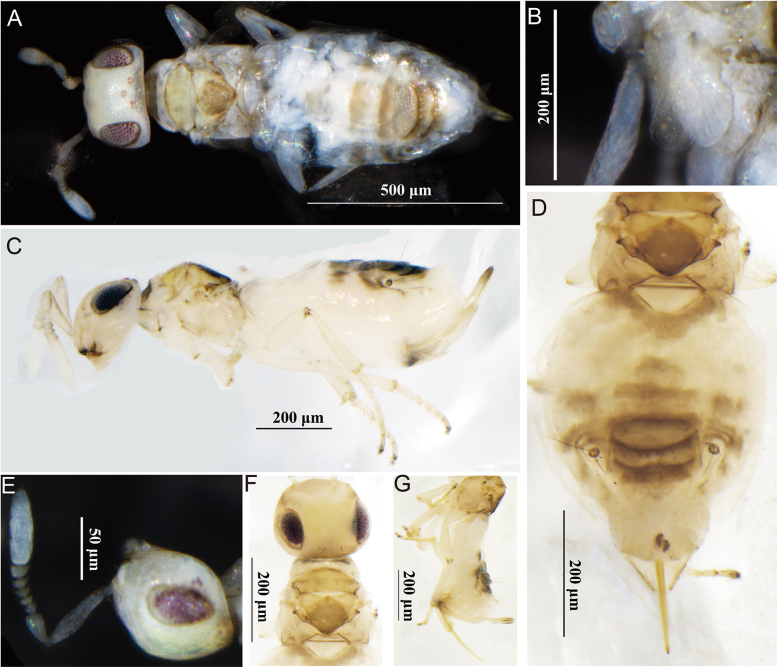
*Acerophagusalbuscorpus* Xi & Hu, sp. nov., holotype female **A** dorsal habitus **B** fore wing **C** lateral habitus **D** mesosoma and metasoma in dorsal view **E** antenna **F** head and mesosoma in dorsal view **G** gaster in lateral view.

##### Description.

**Female** (holotype). ***Body***. Length 1.04 mm; creamy white with multiple gray-brown spots except head, eyes blackish brown, ocelli white with red outline, antenna white with more or less brown margins on all segments except for F1, F2 and clava; pronotum with trapezoidal, gray-brown patch; mesoscutum and axilla yellowish, slightly glossy; scutellum mostly gray-brown; fore wing hyaline; the bases of femora, tibiae and all tarsi of hind legs slightly blackish brown; gaster with grayish-brown transverse band at base of Gt1, middle, and sides of Gt2–7; ovipositor yellowish brown, except base and tip brown.

***Head*.** Dorsal length about half of head width; frontovertex 0.5× head width; occipital margin sharp; ocellus with apical angle obtuse, AOL: OOL: OCL = 3:3.1:2; eye height 1.2× malar space, width slightly equal to gena length. Antennal toruli below the eye lower margin; scape slightly subcylindrical, 4× as long as wide; pedicel slightly conic, short than funicle; F1–5 broader than long, each gradually widen; clava three segments, 3.5× as long as wide and 1.5× as long as funicle. Measurements (μm): head width, 27; frontovertex, 14.5; AOL, 3; OOL, 3.1; OCL, 2; eye length, 29; malar space, 10; gena length, 8; scape length and width, 14 and 3.5; pedicel length, 6; funicle length, 9; clava length and width, 14 and 4.

***Mesosoma***. Mesoscutum slightly swollen, straight posteriorly, about 0.6× as long as wide; axillae large and slightly separated in the middle; scutellum about as long as wide; fore wing strongly reduced, very short and small, about 0.8× as long as broad, not reaching base of gaster; mesotibial spur 0.7× mesotibia length. Measurements (μm): mesosoma length, 28; mesoscutum length, 18; scutellum length and width, 12 and 12; mesotibial spur length, 8; mesotibia length, 22.8; mesobasitarsus length, 8; fore wing length and width, 14.4 and 11.1.

***Metasoma*.** Metasoma slightly < 1.8× mesosoma length; ovipositor exserted, its full length 1.5× as long as mid tibia and 0.7× as long as metasoma. Measurements (μm): metasoma length, 49; ovipositor length, 35.

***Variation*** (female paratypes). Body length 0.84–1.04 mm; eye 1.2–1.3× as long as malar space; antennal clava 3.0–3.5× as long as wide.

**Male.** Unknown.

##### Etymology.

The name of the new species is derived from the Latin words *albus*, “white”, and *corpus*, “body”, and refers to the body color.

##### Comments.

The new species belongs to *Acerophagus* Smith, 1880. The new species is more similar to *Acerophagusovaliclavus* Zu & Li, 2016. However, it differs from *A.ovaliclavus* in the following: frontovertex about 0.5× head wide, scape about 4× as long as broad, clava about 3.5× as long as broad, forewing brachypterous, all gastral terga with grayish-brown transverse bands, ovipositor about 1.5× as long as mid tibia in *A.ovaliclavus*: frontovertex 0.4× head wide, scape about 3.4× as long as broad and clava about 1.6×, forewing normal, only Gt5–7 with grayish-brown transverse bands, ovipositor 1.9× as long as mid tibia (Zu and Li 2017; Hu et al. 2019).

#### ﻿ Pseudectroma stemmatosteres

Taxon classificationAnimaliaHymenopteraEncyrtidae

Zhang & Hu sp. nov.

4FBDAE9B-205C-5E9D-8F29-81482C564A6F

https://zoobank.org/DB28E898-A5BE-4E91-A34A-27B135AF6CE3

Figs 2 , 3

##### Type material.

***Holotype*** • ♀ card mounted, China, Xinjiang, Tashkurgan Tajik Autonomous County, Kokyar Kirghiz Township. 37°44'2.418"N, 75°15'23.4648"E, altitude: 3086 m, 22. VII. 2022. Coll. Hu Hongying’s insect research team by yellow pan trapping. ***Paratypes*** • 4 ♀♀, 5 ♂♂, same data as the holotype (all deposited in ICXU).

**Figure 2.**
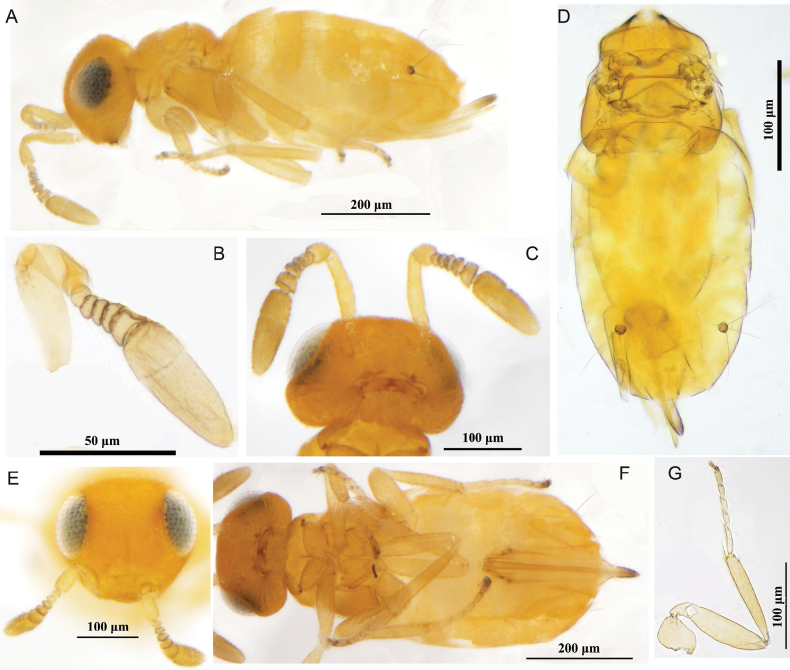
*Pseudectromastemmatosteres* Zhang & Hu, sp. nov., holotype female **A** lateral habitus **B** antenna **C** head in ventral view and antennae **D** mesosoma and metasoma in dorsal view **E** head in frontal view and antennae **F** habitus in ventral view **G** hind leg.

##### Description.

**Female** (holotype). ***Body*.** Length 0.76 mm; yellow, except head orange, eye greyish black, antenna yellowish; mesosoma alutaceous, legs yellow with telotarsus brown; gaster yellowish except ovipositor terminal yellow brown.

**Figure 3.**
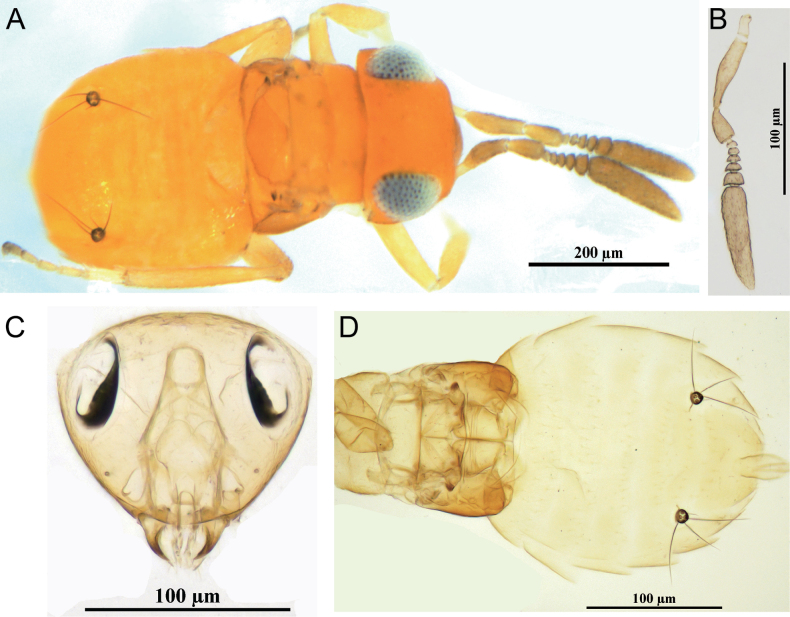
*Pseudectromastemmatosteres* Zhang & Hu, sp. nov., paratype male **A** habitus in dorsal view **B** antenna **C** head in frontal view **D** mesosoma and gaster in dorsal view.

***Head*.** In frontal view, about 1.1× as wide as high and with extremely shallow scaly reticulations; frontovertex about 0.8× head width; in dorsal view, occipital margin bluntly rounded, with posterior margin concave forward; ocelli absent; eyes small, 0.6× head high; toruli below lower margin of eyes and about 0.8× as long as wide. Antenna with scape 3.7× as long as wide and about 0.9× frontovertex width; pedicel about equal to F1–4 combined; all funicle segments transverse, gradually increasing from F1–5; clava 2-segmented, slightly longer than scape and 2.8× as long as wide; mandible tridentate. Measurements (μm): head dorsal width and height, 25 and 22; frontovertex, 13; eye length, 13; antennal scape length and width, 11 and 3; pedicel length and width, 5 and 2.2; funicle length, 8; clava length and width, 11 and 4.

***Mesosoma*.** Pronotum slightly longer than mesoscutum; mesoscutum transverse, 0.37× as long as wide; axilla not separated; scutellum 0.58× as length as width; wings absent; mesotibial spur 0.8× of mesobasitarsus length and 0.3× of mesotibial length. Measurements (μm): pronotum length, 9; mesosoma length, 26; mesoscutum length and width, 7 and 19; scutellum length and width, 7 and 12; mesotibial spur length, 5; mesotibia length, 20; mesobasitarsus length, 6.

***Metasoma*** about 1.9× as long as mesosoma; hypopygium extending almost to apex of gaster; ovipositor exsertedabout 0.6× as long as gaster or 1.5× as long as mesotibia. Gt5 with three long setae on each side. Measurements (μm): metasoma length, 50; ovipositor length, 30.

***Variation*** (female paratypes). Body length, 0.75–0.87 mm; head in frontal view almost 0.9× as broad as high; frontovertex about 0.5× head wide; eye length 0.5–0.6× as long as head height; metasoma 0.3–0.4× as long as wide.

**Male.** Body length 0.64–0.84 mm (Fig. 3). Generally similar to female except for antenna clava longer than female, male clava 3.4× as long as wide. Metasoma nearly round, about 1.1× as long as wide. Other characteristics similar to those in females.

##### Host.

Unknown.

##### Etymology.

The new species name is derived from stemma refers to ocelli.

##### Comments.

In *Pseudectroma* Girault, 1915, 13 species were previously known, but this new species differs from all of these. This new species is most similar to *P.caribe* Noyes, 2010, from which it differs in having the frontovertex about 0.5× the head wide, the occipital margin bluntly rounded, and the ovipositor 1.5× as long as the mesotibia; in *P.caribe*, the frontovertex is about 0.4× the head wide, the occipital margin is acute, and the ovipositor is about 2.0× as long as the mesotibia (Noyes 2010).

### ﻿Species newly recorded from China

#### ﻿ Ericydnus novosibiricus

Taxon classificationAnimaliaHymenopteraEncyrtidae

Sugonjaev & Gavrilyuk, 2012

376399CA-7F95-5CB7-AF2F-7E2FCCCA307D

Fig. 4

Ericydnus novosibiricus Sugonjaev & Gavrilyuk, 2012: 160–163.

##### Material examined.

China, Xinjiang • 5 ♀♀, Tashkurgan Tajik Autonomous County, Wahanzoulang, 37°7'57.59"N, 75°7'5.6892"E, altitude: 3826 m, 23.VII.2021. China, Xinjiang • 3 ♂♂, Dabudaer township, 38°7'50.7864"N, 74°58'39.018"E, altitude: 3566 m, 24.VII.2021. Coll. Hong-Ying Hu group, by sweeping (all deposited in ICXU).

**Figure 4.**
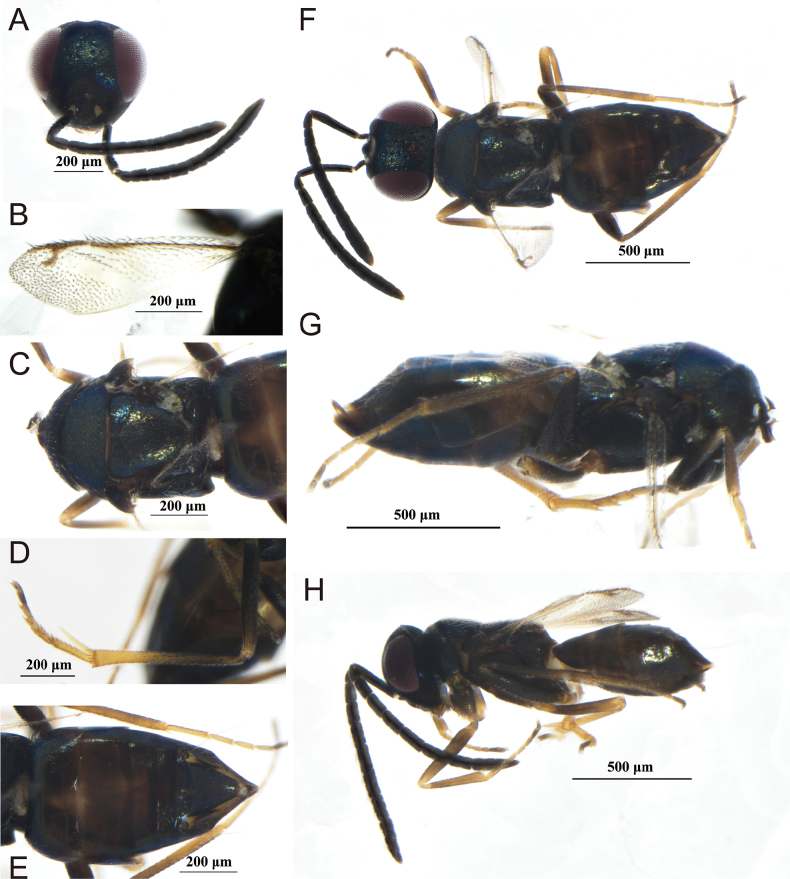
*Ericydnusnovosibiricus* Sugonjaev & Gavrilyuk, female **A** head in frontal view and antennae **B** fore wing **C** mesosoma in dorsal view **D** middle leg **E** gaster in dorsal view **F** dorsal habitus **G** mesosoma and metasoma in lateral view **H** lateral habitus.

##### Diagnosis.

**Female.** Body length about 1.7 mm; body black with blue-green metallic sheen. Eye deep reddish brown; gena with purple metallic luster; antenna and tegula black. Head 1.1× as wide as high; frontovertex about 0.4× head wide, with dense punctuations. Antenna with scape 6.5× as long as wide; pedicel 1.8× as long as wide, slightly shorter than F1; F1–6 slightly longer than wide; clava 3.2× as long as wide. Mesosoma with many rows of white setae; mesoscutum with scaly reticulation, 0.5× as long as wide. Fore wing short, 3.2× as long as wide, only reaching the basal 1/3 of gaster, metasoma longer than mesosoma; hypopygium extending to apex of gaster.

**Male.** Unknown.

##### Host.

Unknown.

##### Distribution.

China (Xinjiang); Russia (Novosibirsk).

##### Comments.

This newly collected specimens match the original description of *E.novosibiricus* (Sugonyaev and Gavrilyuk 2012). However, there are some differences: female body length about 1.7 mm (1.5–1.6 mm in original description); fore wing short and only reaching the basal 1/3 of gaster (considerably not reaching apex of abdomen, but approximately reaching level of pygostyli in original description). Nevertheless, we are confident our specimens are conspecific with *E.novosibiricus*.

#### ﻿ Copidosoma charon

Taxon classificationAnimaliaHymenopteraEncyrtidae

Guerrieri & Noyes, 2005

E8A03053-5680-553F-A428-16739F01511E

Fig. 5

Copidosoma charon Guerrieri & Noyes, 2005: 146.

##### Material examined.

China, Xinjiang • 2 ♀♀, Kizilsu Kirgiz Autonomous Prefecture, Wuqia County, 40°15'6.244"N, 75°29'6.7308"E, altitude: 2765 m, 18.VII. 2021. Coll. Hong-Ying Hu group, by sweeping; China, Xinjiang • 4 ♀♀, Tashkurgan Tajik Autonomous County, Seritashike ranches, 37°25'4.0152"N, 75°22'13.9512"E, altitude: 3394 m, 23.VII.2021. Coll. Jin-Zhe Li, by sweeping (all deposited in ICXU).

**Figure 5.**
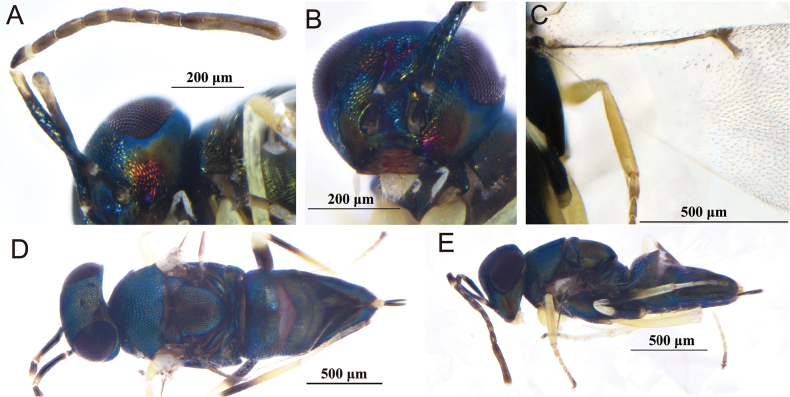
*Copidosomacharon* Guerrieri & Noyes, 2005 female **A** antenna **B** head in frontal view **C** forewing **D** dorsal habitus **E** lateral habitus.

##### Diagnosis.

**Female.** Body length about 1.8 mm; body with blue-green metallic sheen; antennal scape with black metallic sheen, pedicel and flagellum brown. Frontovertex about 0.5× head width; malar space almost 0.7× as long as eye, slightly shorter than eye width. Antenna scape elongated, about 9.8–10.2× as long as wide, funicle 6-segmented, clava 3-segmented, about 5.5–6.1× as long as wide. Mesoscutum with regular, elongated sculpture; fore wing slender, 2.3× as long as wide; mesotibial spur 0.9× of mesobasitarsus length and about 0.25× of mesotibia length. Metasoma and mesosoma almost equal in length; hypopygium extending to apex of gaster; ovipositor about 0.2× as long as gaster.

**Male.** Unknown.

##### Host.

Unknown.

##### Distribution.

China (Xinjiang); Austria, Bosnia and Herzegovina, Czech Republic, UK (England), Finland, France, Spain, Sweden.

##### Comments.

Our specimens conform to original description of *C.charon* except for some minor differences: antenna scape more elongate, about 9.8× as long as wide, (7.5× as long as wide in the original description); fore wing 2.3× as long as wide (about 2.5 as long as broad in original description). We are convinced that our specimens are conspecific with *C.charon* (Guerrieri and Noyes 2005).

#### ﻿ Trichomasthus marsus

Taxon classificationAnimaliaHymenopteraEncyrtidae

(Walker, 1837)

0EAC3D66-018D-55A7-94E6-F7A2CD19C485

Fig. 6

Encyrtus marsus Walker, 1837: 444.
Trichomasthus marsus : Graham 1959: 147–175.

##### Material examined.

China, Xinjiang • 8 ♀♀, Kizilsu Kirgiz Autonomous Prefecture, Aketao County, 38°22'27.876"N, 74°59'54.0312"E, altitude: 3664 m, 19. VII. 2021. Coll. Hong-Ying Hu group, by sweeping (all deposited in ICXU).

**Figure 6.**
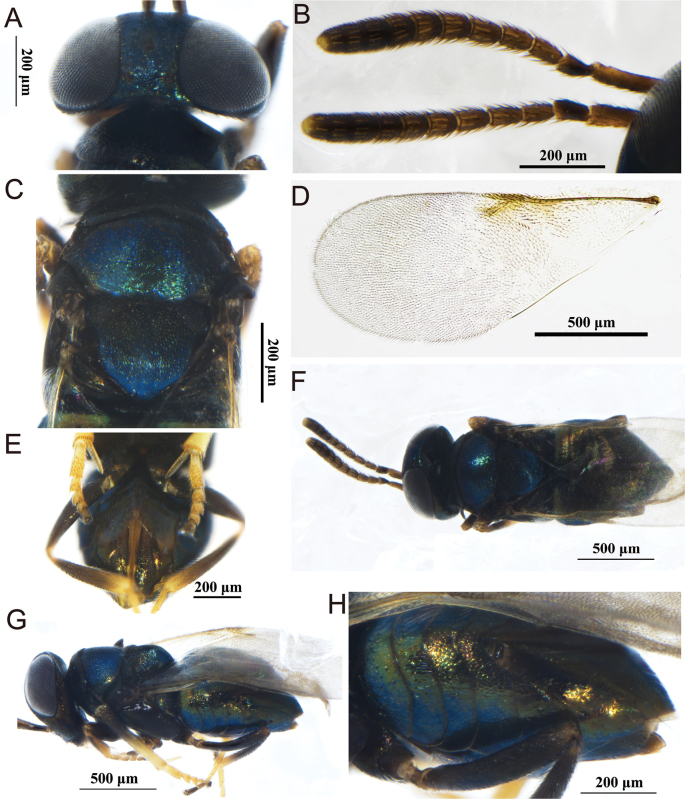
*Trichomasthusmarsus*, female **A** head in dorsal view **B** head in ventral view **C** mesosoma in dorsal view **D** fore wing **E** hypopygium **F** dorsal habitus **G** dorsolateral habitus **H** gaster in lateral view.

##### Diagnosis.

**Female.** Body length 1.1–1.3 mm; head dark brown with slightly metallic sheen; antenna with scape black, funicle yellowish brown, clava blackish brown. Mesonotum with blue-green strong metallic tinge; axilla and scutellum dark brown; fore and mid legs yellow and hind leg black. Frontovertex at most 1/3 head wide; scape slender, about 5× as long as wide; pedicel slightly longer than F1; pronotum and mesoscutum with reticulate sculpture. Fore wing hyaline, 1.8× as long as wide, marginal vein 3× as long as wide, marginal vein as long as postmarginal vein; stigma vein slightly elongated; stigma vein slightly longer than postmarginal vein. Gaster triangular and about equal to mesonotum length, hypopygium extending to middle of gaster.

**Male.** Unknown.

##### Host.

Unknown.

##### Distribution.

China (Xinjiang); Germany, Norway, United Kingdom.

##### Comments.

Our newly recorded female specimen matches the original description of *T.marsus* (Jensen and Sharkov 1989). However, it differs in the following: funicle yellowish brown and clava blackish brown (both funicle and clava blackish brown in original description); stigma vein slightly longer than postmarginal vein (stigma vein as long as postmarginal vein in original description). The other characteristics are consistent with the original description.

#### ﻿ Aphycus slavai

Taxon classificationAnimaliaHymenopteraEncyrtidae

Myartseva, 1981

3F6AC537-0C47-5C82-8A71-2791906AD9D8

Fig. 7

Aphycus slavai Myartseva, 1981d: 20–21.
Aphycus slavai Myartseva: Japoshvili 2016: 3; Japoshvili 2017a: 3.

##### Material examined.

China, Xinjiang • 8 ♀♀, 3 ♂♂, Tashkurgan Tajik Autonomous County, Kashgar, 38°7'50.7864"N, 74°58'39.018"E, altitude: 3566 m, 24. VII. 2021. Coll. Hong-Ying Hu group, by sweeping (all deposited in ICXU).

##### Diagnosis.

Female. Body length about 1.1 mm; body dark brown, except head yellow, mesosoma yellow with dark-brown bands, densely covered with white bristles; antennae and legs light brown; fore wing hyaline with brown spots. Head about 0.9–1.0× as wide as high; frontovertex about 0.3× head wide; ocelli arranged in an equilateral triangle; OOL equal to OCL. Antenna inserted significantly below lower ocular line; scape nearly cylindrical, 7.1× as long as wide; pedicel 2.6× as long as wide; F1–4 transverse, F5 and F6 almost square. Mesoscutum about 0.7× as long as wide, with shallow reticulation; scutellum almost as long as wide; fore wing about 2.4× as long as wide; marginal vein absent; postmarginal vein short and about 0.3× as long as stigma vein; mesotibial spur shorter than mesobasitarsus. Hypopygium slightly extending from apex of gaster.

**Figure 7.**
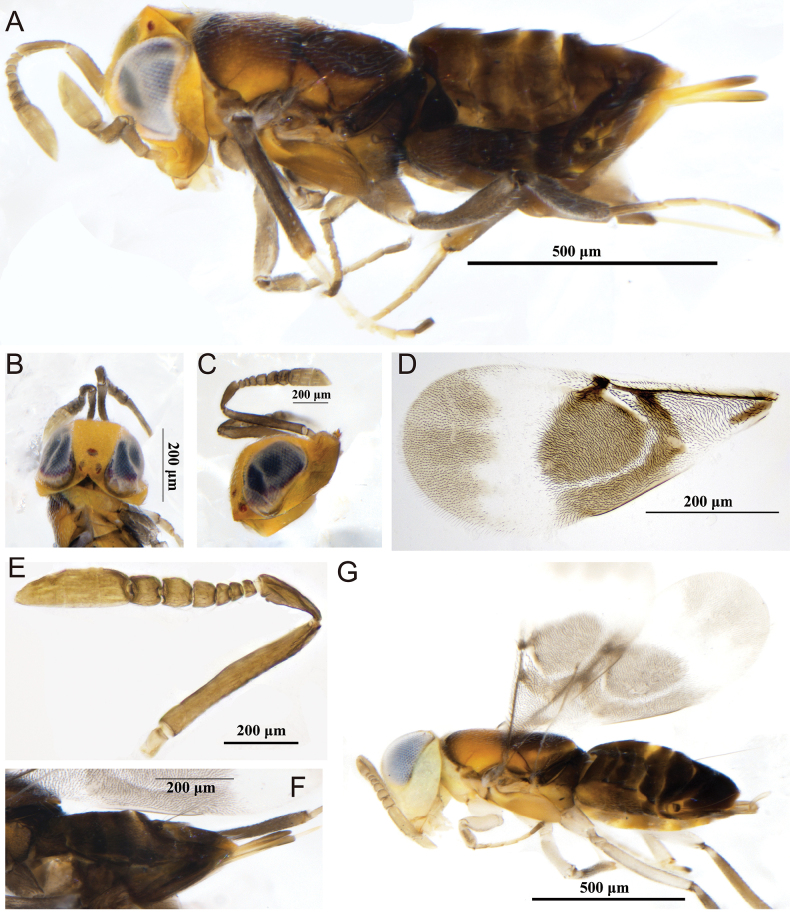
*Aphycusslavai***A–F** female **A** lateral habitus **B** head in dorsal view **C** head in lateral view **D** fore wing **E** antenna **F** gaster in dorsolateral view **G** male, lateral habitus.

**Male.** Mostly similar to female except pedicel relatively shorter and clava unsegment; body paler (Fig. 7G).

##### Host.

*Phenacoccuspersimplex* Borchsenius, 1949 (Hemiptera, Pseudococcidae) (Trjapitzin 1989; Japoshvili et al. 2016).

##### Distribution.

China (Xinjiang); Turkmenistan.

##### Comments.

This species is very similar to *A.sulamanidzei*, but the main difference is in the color of the abdomen; in *A.sulamanidzei* the entire abdomen is yellow. Our specimen matches the characteristics of *A.slavai* in having a dark-brown abdomen. Other characteristics are consistent with the original description of *A.slavai* (Myartseva 1981).

## ﻿Discussion

We surveyed the eastern Pamir Plateau for three years, both in core areas and at higher elevations with diverse habitat types. We found 15 genera and 21 species. These taxa are likely to be better adapted to alpine environments.

This study primarily adds to our knowledge of the biodiversity of the family Encyrtidae on the eastern Pamir Plateau. Two new species of encyrtids are identified and essential data are provided for the biological management of agroforestry pests in alpine environments and the protection of natural pest insects. *Copidosomatruncatellum* was most abundant species found and was mostly distributed in Aktao and Wuqia counties. However, we used net-scanning to collect specimens, so very little information was gathered on the hosts of the species found. In the future, we aim to collect hosts and perform lab rearing to clarify hosts. For the two new species, few specimens were collected, and we will try to collect additional material.

## Supplementary Material

XML Treatment for Acerophagus albuscorpus

XML Treatment for Pseudectroma stemmatosteres

XML Treatment for Ericydnus novosibiricus

XML Treatment for Copidosoma charon

XML Treatment for Trichomasthus marsus

XML Treatment for Aphycus slavai

## ﻿Additional information

### Author ORCIDs

Ouyan Xi https://orcid.org/0009-0002-7039-5952

Hongying Hu https://orcid.org/0000-0002-2295-1072

### Data availability

All of the data that support the findings of this study are available in the main text.
